# JAK–STAT signaling pathway in the pathogenesis of atopic dermatitis: An updated review

**DOI:** 10.3389/fimmu.2022.1068260

**Published:** 2022-12-08

**Authors:** I-Hsin Huang, Wen-Hung Chung, Po-Chien Wu, Chun-Bing Chen

**Affiliations:** ^1^ Drug Hypersensitivity Clinical and Research Center, Chang Gung Memorial Hospital, Linkou, Taipei, and Keelung, Taoyuan, Taiwan; ^2^ Department of Dermatology, Chang Gung Memorial Hospital, Linkou, Taipei and Keelung, Taiwan; ^3^ Research Center of Big Data and Meta-analysis, Wan Fang Hospital, Taipei Medical University, Taipei, Taiwan; ^4^ Cancer Vaccine and Immune Cell Therapy Core Laboratory, Chang Gung Memorial Hospital, Linkou, Taiwan; ^5^ Chang Gung Immunology Consortium, Chang Gung Memorial Hospital, Linkou, and Chang Gung University, Taoyuan, Taiwan; ^6^ Department of Dermatology, Xiamen Chang Gung Hospital, Xiamen, China; ^7^ Xiamen Chang Gung Allergology Consortium, Xiamen, Xiamen Chang Gung Hospital, Xiamen, China; ^8^ College of Medicine, Chang Gung University, Taoyuan, Taiwan; ^9^ Whole-Genome Research Core Laboratory of Human Diseases, Chang Gung Memorial Hospital, Keelung, Taiwan; ^10^ Immune-Oncology Center of Excellence, Chang Gung Memorial Hospital, Linkou, Taiwan; ^11^ Genomic Medicine Core Laboratory, Chang Gung Memorial Hospital, Linkou, Taiwan; ^12^ Graduate Institute of Clinical Medical Sciences, College of Medicine, Chang Gung University, Taoyuan, Taiwan; ^13^ School of Medicine, National Tsing Hua University, Hsinchu, Taiwan

**Keywords:** atopic dermatitis (AD), Janus kinase (JAK), Janus kinase inhibitor, signal transducer and activator of transcription (STAT), review

## Abstract

Atopic dermatitis (AD) is a chronic, inflammatory, pruritic form of dermatosis with heterogeneous manifestations that can substantially affect patients' quality of life. AD has a complex pathogenesis, making treatment challenging for dermatologists. The Janus kinase (JAK)–signal transducer and activator of transcription (STAT) pathway plays a central role in modulating multiple immune axes involved in the immunopathogenesis of AD. In particular, Th2 cytokines, including interleukin (IL)-4, IL-5, IL-13, IL-31, and thymic stromal lymphopoietin, which contribute to the symptoms of chronic inflammation and pruritus in AD, are mediated by JAK–STAT signal transduction. Furthermore, JAK–STAT is involved in the regulation of the epidermal barrier and the modulation of peripheral nerves related to the transduction of pruritus. Targeting the JAK–STAT pathway may attenuate these signals and show clinical efficacy through the suppression of various immune pathways associated with AD. Topical and oral JAK inhibitors with variable selectivity have emerged as promising therapeutic options for AD. Notably, topical ruxolitinib, oral upadacitinib, and oral abrocitinib were approved by the U.S. Food and Drug Administration for treating patients with AD. Accordingly, the present study reviewed the role of JAK–STAT pathways in the pathogenesis of AD and explored updated applications of JAK inhibitors in treating AD.

## 1 Introduction

Atopic dermatitis (AD), a chronic inflammatory dermatosis, clinically manifests as eczematous and itchy skin lesions with a predictable distribution on the basis of the patient’s age (1, 2). Chronic pruritus may result in sleep disturbance, decreased quality of life, low self-esteem, and a higher risk of anxiety or depression (3, 4). Long-term inflammation could result in lichenification and has been linked to frequent cutaneous infection, particularly *Staphylococcus aureus* colonization (5, 6). AD affects up to 20% of children, especially in families with atopic history, and it affects 10% of adults, although the rate varies by region (7–9). The interaction between genetic, environmental, and immunological factors was implicated in the pathogenesis of AD (10). AD comprises intrinsic and extrinsic variations and can be divided into subtypes on the basis of filaggrin mutation status, patient race, and age (11). Clinical manifestations and molecular profiles may vary from different endotypes. The immunopathogenesis of AD involves the interplay of innate and adaptive immune responses (12). The innate immune responses in AD are initiated from epithelial defects or defects in the expression levels of specific proteins or innate receptors, resulting in the stimulation of the adaptive immune system, and type 2 helper T (Th2) cells in particular (13, 14). The complex immune axes involved in AD complicate treatments with conventional medicines. Nevertheless, emerging biologics and small molecules have brought a new era in AD treatment (15, 16). Small molecules that target the Janus kinases (JAK) signal mediators, which regulate the proinflammatory cytokines, constitute a promising treatment for AD. Accordingly, this study reviews the role of the JAK–Signal Transducer and Activator of Transcription (STAT) pathway in the pathogenesis of AD and provides updated data supporting the applicability of JAK inhibitors in treating AD.

## 2 Role of the JAK–STAT pathway in the pathogenesis of AD

### 2.1 Pathogenesis of AD

The development of AD involves complex multifactorial pathophysiologies, comprising an impaired skin barrier, immunoglobulin E (IgE) sensitization, alterations of type 2 immune responses to cellular infiltration, and environmental and genetic factors (17). Dysfunction of the skin barrier is postulated to be a cause and a consequence of AD. Skin barrier abnormalities could be caused by a mutation of the filaggrin (FLG) gene, which encodes the key barrier protein FLG; irregularities of other structural proteins or lipids; or dysfunction of keratinocytes. Any of these abnormalities could lead to an impaired antimicrobial effect and a higher risk of antigen penetration (18). The disruption of the skin barrier triggers keratinocytes to release thymic stromal lymphopoietin (TSLP), which is a Th2-type immune-inducing factor (19). TSLP is also secreted by stromal cells, epithelial cells, fibroblasts, keratinocytes, and basophils, and is highly associated with other inflammatory diseases (20).

**Figure 1:**
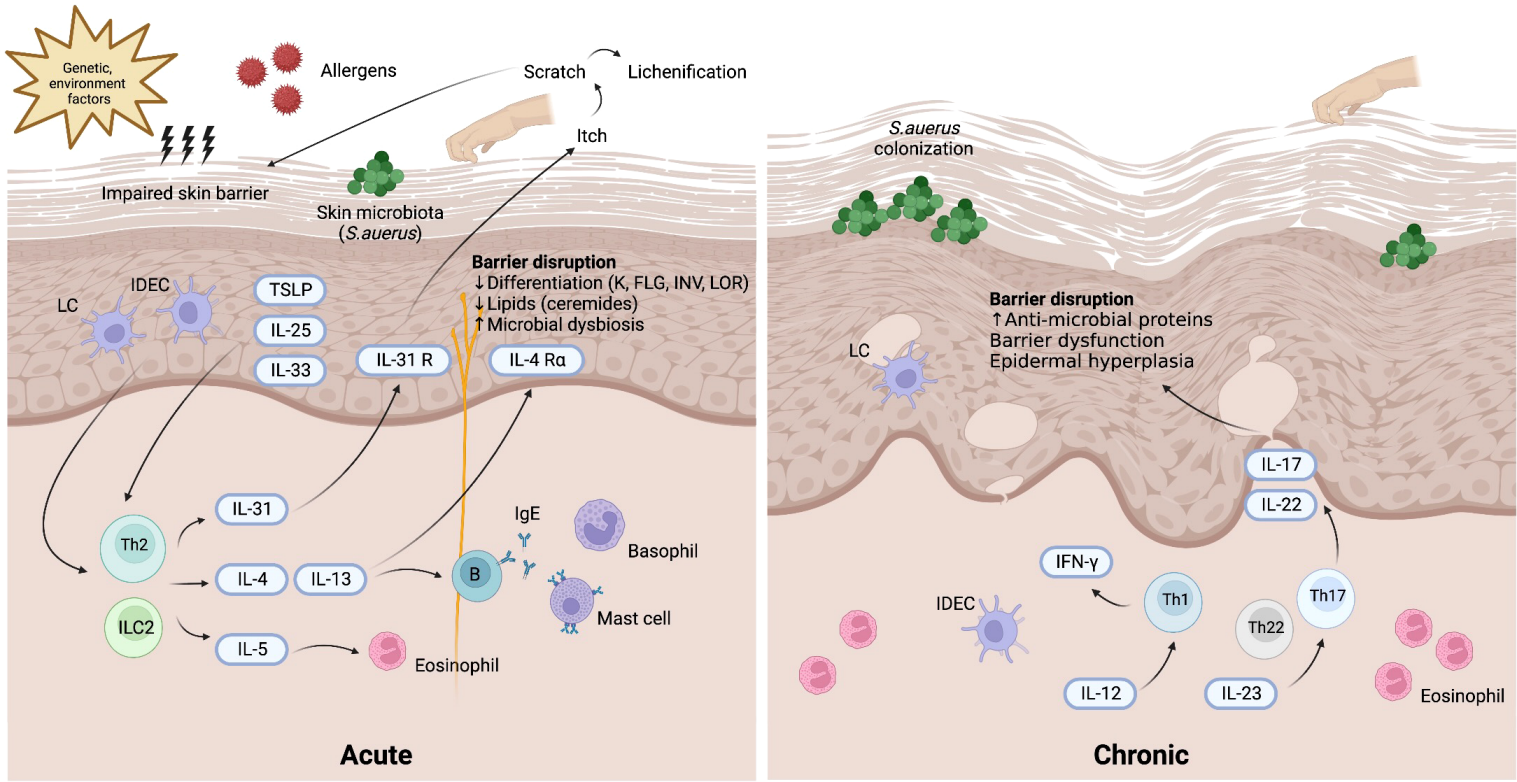
Pathogenesis of atopic dermatitis. Background genetic predisposition and environment factors may induce skin barrier impairment along with the invasion of allergens and skin microbiota (mostly *Staphylococcus aureus*). TSLP, IL-25, and IL-33 released from keratinocytes and activated skin LCs and IDECs lead to the activation of Th2 axis immune response through ILC2 and Th2 cells. In the acute phase of atopic dermatitis, the production of various Th2 cytokines including IL-4, IL-5, IL-13, IL-31 results in barrier dysfunction. In the chronic phase of atopic dermatitis, Th17 and Th22 cytokines cooperatively modulate local inflammation through upregulation of proinflammatory cytokines stimulating epidermal hyperplasia. Th1 immune response also plays a role in the chronic phase of atopic dermatitis. IDEC, inflammatory dendritic epidermal cell; IFN, interferon; IL, interleukin; ILC2, type 2 innate lymphoid cell; LC, Langerhans cell; R, receptor; *S. aureus*, *Staphylococcus aureus*; TSLP, thymic stromal lymphopoietin.

The immunopathogenesis of AD is suggestive of a biphasic T-cell-mediated inflammatory disease, wherein a Th2 immune response is responsible for the initial acute stage, and then the Th1 immune axis takes over as the disease progresses into its chronic stage (1) (**Figure 1**). In addition to Th cells, the presence of cytotoxic T cells, dendritic cells, eosinophils, and mast cells is markedly increased in AD skin lesions relative to healthy skin. During the acute phase, Th2-predominant immune responses trigger the release of Th2 cytokines such as interleukin (IL)-4, IL-5, IL-13, and IL-31 to promote local inflammation after stimulation by allergens, TSLP or IL-33. As the central cytokines in the pathogenesis of AD, IL-4 and IL-13 activate and promote Th2 cells, induce differentiation and activation of myeloid and atopic dendritic cells, activate B cells, stimulate IgE class-switching, and recruit eosinophils (21). Clinically, IL-4 and IL-13 contribute to skin barrier defects, cutaneous infections, inflammation, skin thickening, and itchiness (22). IL-4 and IL-13 are also associated with AD disease activity (23). As for IL-31, it is postulated to account for the symptom of pruritus in AD (24). Additionally, IL-31 delays the apoptosis of eosinophils through the expression of IL-31 receptor A IL 31RA on the surface of eosinophils (25). Another Th2 cytokine, IL-5, is a key mediator of eosinophil differentiation and proliferation in the bone marrow (26). Additionally, IL-5 can activate and prime eosinophils, increasing their sensitivity to allergic inflammatory reactions (27). In the chronic phase of AD, increased expression levels of IL-12, interferon-gamma (IFN-γ), and granulocyte–macrophage colony-stimulating factor (GM-CSF) are characteristic of a Th1 axis immune response (1). Additionally, Th17 and Th22 cytokines (IL-17, IL-19, and IL-22) contribute to the formation of chronic skin lesions in AD, especially in pediatric, Asian, and genetically predisposed patients (28). In contrast to the Th1 and Th2 immune responses, Th17 produces IL-17A, IL-17F, IL-22, and tumor necrosis factor-alpha (TNF-α). These cytokines promote cell proliferation and macrophage infiltration into damaged skin and stimulate eosinophils to secrete profibrotic cytokines (29). Although IL-17 is not as predominant in AD as it is in psoriasis, studies have revealed that IL-17 can stimulate the differentiation of B cells into IgE-producing plasma cells, promote the release of IL-8, TNF-α, and TSLP, activate keratinocytes to express adhesion molecules, and modulate the Th2 immune response, thus triggering the chronic phase of AD (29–31). Furthermore, T cells can promote keratinocyte hyperplasia and the production of antimicrobial peptides through IL-22 (32).

### 2.2 JAK-STAT pathway

The JAK–STAT signaling pathway is essential to the downstream regulation of various cytokines and growth factors that contribute to a diverse array of biological developmental and homeostatic processes, cell proliferation, and immune regulation. The JAK kinase family comprises four receptor-associated kinases: JAK1, JAK2, JAK3, and TYK2 (tyrosine kinase 2), and the STAT family comprises seven proteins: STAT1, STAT2, STAT3, STAT4, STAT5A, STAT5B, and STAT6 (33). Generally, the JAK–STAT signal cascade is initiated by the ligation of extracellular cytokines with their cognate receptors. JAKs bind directly to the intracellular domains of type I and II cytokine receptor chains (**Table 1**) (34, 35), resulting in the apposition of the JAKs and the transphosphorylation and activation of the receptors. Accordingly, activated JAKs may phosphorylate tyrosine residues on the cytokine receptors and on other JAKs to create docking sites, thereby enabling the recruitment and phosphorylation of principal downstream signaling molecules such as STAT proteins (36). Once phosphorylated, the STATs dimerize and subsequently translocate into the nucleus, where they bind to DNA and regulate gene transcription (33). Different JAKs and STATs are recruited into this process according to the tissue specificity and the receptors involved in the signaling event (37).

**Table 1:** Families of interleukin transduction signals *via* the JAK–STAT pathway.

Classification	Cytokines	JAKs	STATs
Class I cytokine signaling			
IL-2 family	IL-2, IL-4 (Type I), IL-7, IL-9, IL-15, IL-21	JAK1, JAK3	STAT1, STAT3, STAT5, STAT6
	IL-4 (Type II), IL-13	JAK1, TYK2	STAT6
IL-3 family	IL-3, IL-5, GM-CSF	JAK2	STAT5
IL-6 family	IL-6, IL-11, IL-31, LIF, CNTF, CT1, CLC, OSM	JAK1*	STAT1, STAT3, STAT6
IL-12/23	IL-12, IL-23	TYK2/JAK2	STAT5
Homodimeric (tall)	LEP	JAK2	STAT5
	G-CSF	JAK1*	STAT3
Homodimeric (short)	TPO, EPO, GH, PRL	JAK2	STAT5
Class II cytokine signaling			
Type I IFN	IFN-α, IFN-β, IFN‐ϵ, IFN‐κ, and IFN‐ω	JAK1, TYK2	STAT1, STAT2
Type II IFN	Interferon-γ	JAK1, JAK2	STAT1
Type III IFN	IFN‐λ1 (IL-29), IFN‐λ2 (IL-28A), and IFN‐λ3 (IL-28B)	TYK2, JAK1	STAT1, STAT2
IL-10 family	IL-10, IL-19, IL-20, IL-22, IL-24, IL-26	TYK2, JAK1	STAT3

*JAK1 predominance with assistance of JAK2 and TYK2.

CLC, cardiotrophin-like cytokine; CNTF, ciliary neurotrophic growth factor; CT1, cardiotrophin 1; EPO, erythropoietin; G-CSF, granulocyte colony stimulating factor; GH, growth hormone; GM-CSF, granulocyte/macrophage colony stimulating factor; IFN, interferon; IL, interleukin; JAK, Janus kinase; LEP, leptin; LIF, leukemia inhibitory factor; NK, natural killer; OSM, oncostatin M; PRL, prolactin; STAT, signal transducer and activator of transcription; TPO, thrombopoietin; TSLP, thymic stromal lymphopoietin; TYK, tyrosine kinase.

### 2.3 JAK-STAT signaling pathways in AD

#### 2.3.1 Th2 axis immune response

In AD, epithelial cell–derived cytokines such as IL-25, IL-33, and TSLP promote the overexpression of Th2 cytokines, including IL-4, IL-5, IL-13, and IL-31, either directly through Th2 cytokine-secreting cells or indirectly through dendritic cell-mediated polarization (38). IL-4 performs its biological function by binding to an IL-4 receptor (IL-4R), which is a heterodimeric receptor complex on the cellular surface. IL-4R has two subtypes. Type I IL-4R comprises an IL-4 receptor alpha (IL-4Rα) chain and a common gamma (γ) chain, whereas type II IL-4R comprises an IL-4Rα and an IL-13 receptor alpha 1 (IL-13Rα1) chain (39, 40). IL-13Rα1, along with IL-13 receptor alpha 2 (IL-13Rα2), is a subtype of the IL-13 receptor alpha (IL-13Rα) chain. Unlike the ubiquitously presented IL-4R, IL-13Rα1 chains are expressed on limited cell surfaces including those of B cells, monocytes, macrophages, basophils, mast cells, and endothelial cells (39). Nonhematopoietic structural tissue cells such as keratinocytes also express relatively high amounts of IL-13Rα1, facilitating type II IL-4 receptor formation even with low levels of or absence of common γ chain (γc). Considering IL-4Rα as a functional component responsible for intercellular signal transduction, IL-13 may deliver its signal by binding type II IL-4R with the coexpression of the IL-4Rα and IL-13Rα1 chains (41). Different JAKs bind to different subunits of the receptors, accounting for the variability of JAK–STAT signaling pathways. For example, JAK1, JAK3, and TYK2 bind to IL-4Rα, γc chain, and IL-13Rα subunits of the IL-4R receptors, respectively.

**Figure 2:**
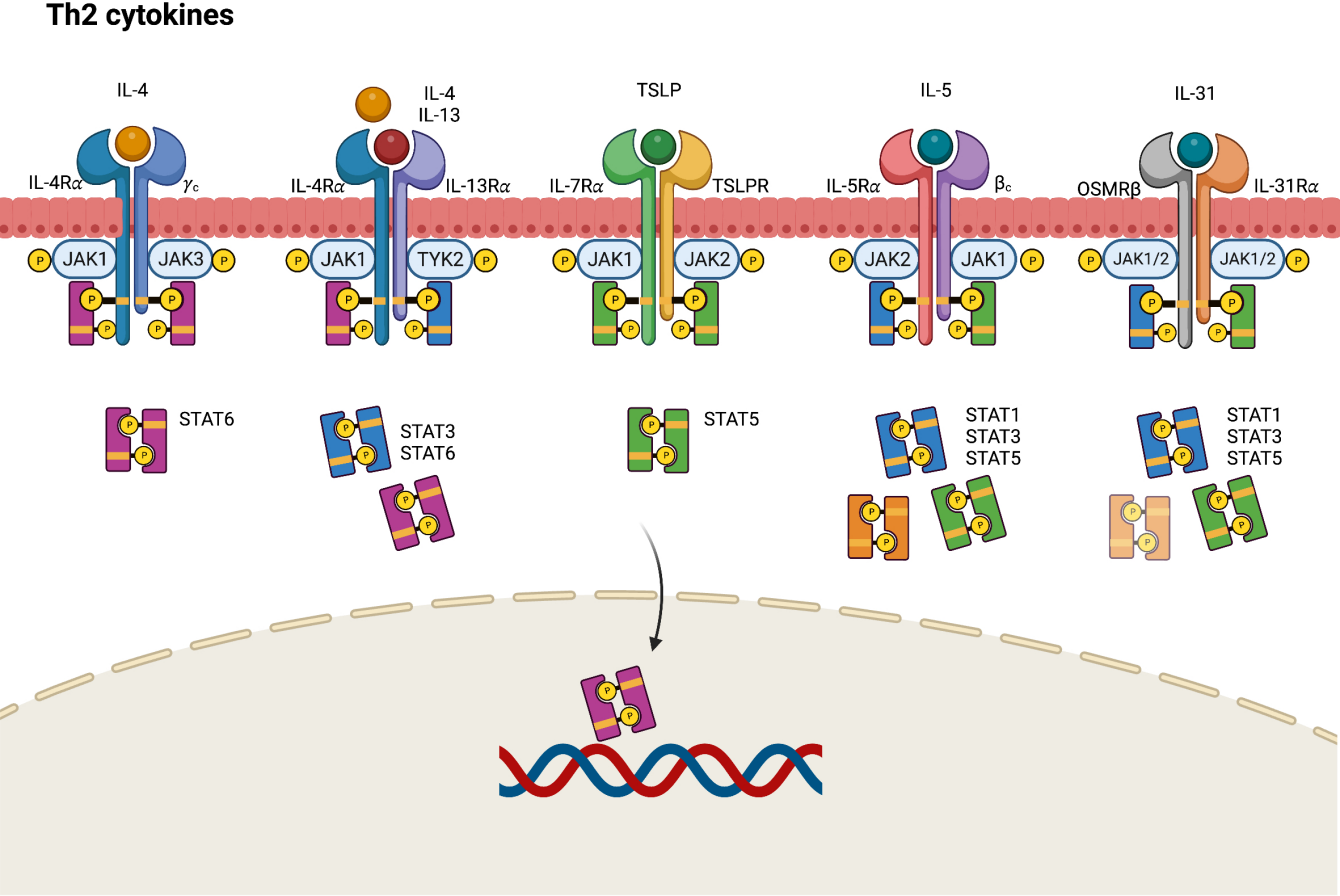
JAK-STAT signaling pathway in Th2 axis immune response of atopic dermatitis. IL, interleukin; JAK, Janus kinase; R, receptor; OSMR, oncostatin M receptor; STAT, signal transducer and activator of transcription; TSLP, thymic stromal lymphopoietin.

In the IL-4 mediated immune response, IL-4 binds to type I IL-4R, stimulating the phosphorylation of JAK1 and JAK3, which in turn activate and phosphorylate IL-4Rα and STAT6 (**Figure 2**) (42). The phosphorylated STAT6 subsequently dimerizes and acts as a transcription factor by binding to specific DNA sequences of IL-4-responsive genes. Activated JAK1 can also phosphorylate Fes kinase, which then phosphorylates insulin receptor substrate-1/2 (IRS-1/2), a protein on the IL-4Rα chain linked to several downstream signaling pathways. Phosphorylated IRS-1/2 recruits p85 regulatory subunits, which activate the phosphatidylinositol 3-kinase (PI3K)–protein kinase B (AKT) pathway. The PI3K–AKT pathway then promotes the proliferation, regeneration, and apoptosis of keratinocytes (43, 44). Conversely, IL-4 and IL-13 can bind to type II IL-4R, inducing the robust phosphorylation of JAK1 and TYK2, followed by the activation and phosphorylation of STAT3 and STAT6. This results in the downregulation of FLG expression and skin barrier dysfunction, and the increased production of TSLP, IL-25, and IL-33 in keratinocytes (45). The IL-4-induced JAK–STAT signal cascade can be inhibited by suppressors of cytokine signaling-1 (SOCS-1), which directly prevent JAK1/STAT6 phosphorylation and arrest IL-4-induced gene expression.

TSLP, a pro-Th2 cytokine, plays a key role in the activation and migration of dendritic cells, which drive the differentiation of Th2 cells to produce IL-4, IL-5, and IL-13 (46). TSLP is highly associated with IL-7, another stromal cell-derived cytokine. TSLP signals by binding to a heterodimeric receptor comprising IL-7 receptor alpha (IL-7Rα) and TSLPR, which interact with JAK1 and JAK2, respectively (20). Accordingly, the TSLP-mediated response activates STAT5 through the phosphorylation of JAK1 and JAK2 (20, 47).

The IL-5 receptor (IL-5R) comprises an alpha subunit (IL-5Rα), which specifically recognizes only IL-5 and a common beta (βc) heterodimeric chain, which can nonspecifically conjugate with not only IL-5 but also IL-3 and granulocyte-macrophage colony stimulating factor (GM-CSF) (27, 48, 49). In the JAK–STAT signaling pathway, the conjugation of IL-5 and the IL-5R triggers the phosphorylation of JAK1 and JAK2, resulting in the activation of STAT1, STAT3, and STAT5 (50).

IL-31 is a helical cytokine belonging to the glycoprotein 130 (gp130)/IL-6 family, a group of receptor proteins that share a common gp130 subunit in their receptor complex (51). IL-31 constitutively engages with the IL-31 receptor (IL-31R), a heterodimeric receptor comprising an IL-31 receptor alpha chain (IL-31Rα) coupled with an oncostatin M receptor beta chain (OSMRβ) (51). The intercellular signals of IL-31 are transmitted by the JAK–STAT pathway, mitogen-activated protein kinases (MAPKs), and the PI3K–AKT pathway. By engaging with the IL-31Rα–OSMRβ complex, IL-31 triggers the activation of JAK1 and JAK2, thereby stimulating STAT3, STAT5 and, to a lesser degree, STAT1 transcriptional activity (52).

#### 2.3.2 Th17/Th22 axis immune response

**Figure 3:**
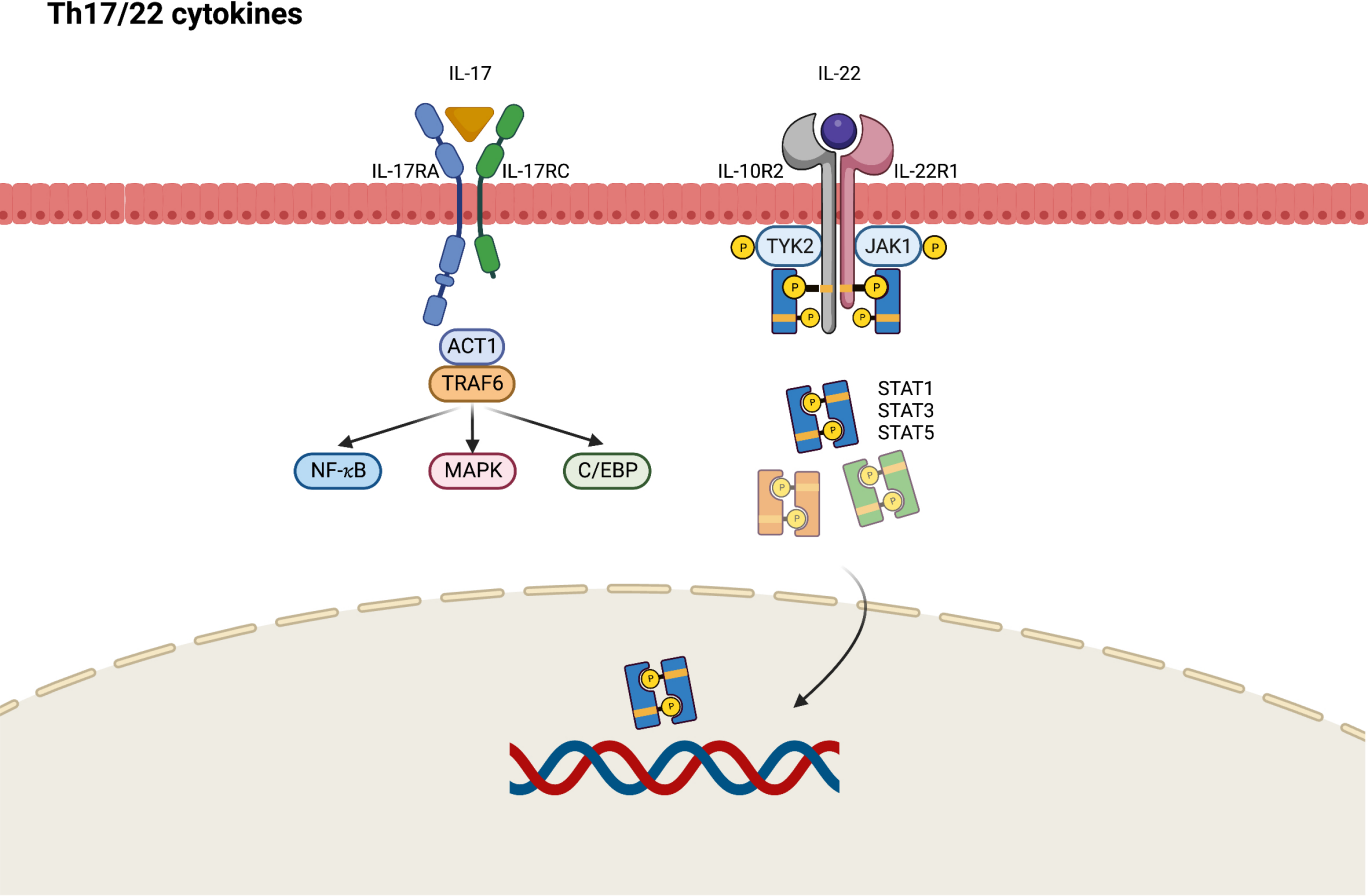
JAK-STAT signaling pathway in Th17/Th22 axis immune response of atopic dermatitis. C/EBP, CCAAT/enhancer binding protein; IL, interleukin; JAK, Janus kinase; MAPK, mitogen-activated protein kinases; NF, nuclear factor; R, receptor; STAT, signal transducer and activator of transcription; TRAF, tumor necrosis factor receptor-associated factors; TYK2, tyrosine kinase 2.

During the chronic phase of AD, Th17 and Th22 cytokines cooperatively modulate local inflammation through upregulation of proinflammatory cytokines that stimulate epidermal hyperplasia (53). IL-17A and IL-17F are eponymous cytokines of the Th17 cell lineage, and IL-17A is believed to have a greater influence on driving autoimmunity than IL-17F because of its more potent signaling ability (54). The IL-17 receptor comprises IL-17RA and IL-17RC, which present different affinities to IL-17A and IL-17F, resulting in different ligand preferences depending on the ratios of IL-17RA and IL-17RC in the receptor complexes (54). In contrast with the classic Th1 and Th2 immune response through the JAK–STAT signaling pathway, Th17-derived IL-17 recruits ACT1, an IL-17 receptor complex adaptor protein, thus engaging with tumor necrosis factor receptor-associated factors (TRAF6) and activating nuclear factor (NF)-κB, CCAAT/enhancer binding protein (C/EBP), and MAPK pathways (**Figure 3**) (54). Generally, it seems that JAK–STAT pathway is not directly involved in the Th17 signaling and may only play a limited role. Nevertheless, STAT3 is found to orchestrate diverse aspects of Th17 differentiation and function (55). *Via* STAT3, a combination of Th17-promoting cytokines including IL-6, IL-21, and IL-23 would stimulate STAT3-dependent Th17 differentiation and enhance the production of IL-17 (56). STAT3 also mediate multiple epigenetic modification of Th17 gene transcription and is essential for Th17 proliferation and survival (57). As a result, selective JAK inhibitors may show the impact on the inhibition of Th17 immune response in AD *via* targeting upstream STAT3 activation.

IL-22 was originally named IL-10-related T-cell-derived inducible factor (IL-TIF) and is identified as a key mediator of epidermal hyperplasia (58). Studies have primarily attributed IL-22 production to Th17- and Th22-cell subsets (59). IL-22 heterodimeric receptor complex comprises the IL-22 receptor (IL-22R1) and IL-10 receptor 2 (IL-10R2) subunits, which are expressed in small amounts by keratinocytes of the skin, gut, and lungs. IL-10R2 is also a subunit of the receptors for IL-10, IL-26, IL-28, and IL-29, whereas IL-22R1 is a component of IL-20 receptor 2 (IL-20R2) for IL-20 and IL-24 (58). After binding to IL-22R1, IL-22 induces the phosphorylation of JAK1 and TYK2. Although STAT3 is considered to be the primary mediator in this cascade, the activation of STAT1 and STAT5 has also been observed in IL-22 signaling (60). Apart from the JAK–STAT pathway, MAPK and p38 pathways are also engaged in IL-22 signaling (58).

#### 2.3.3 Th1 axis immune response

**Figure 4:**
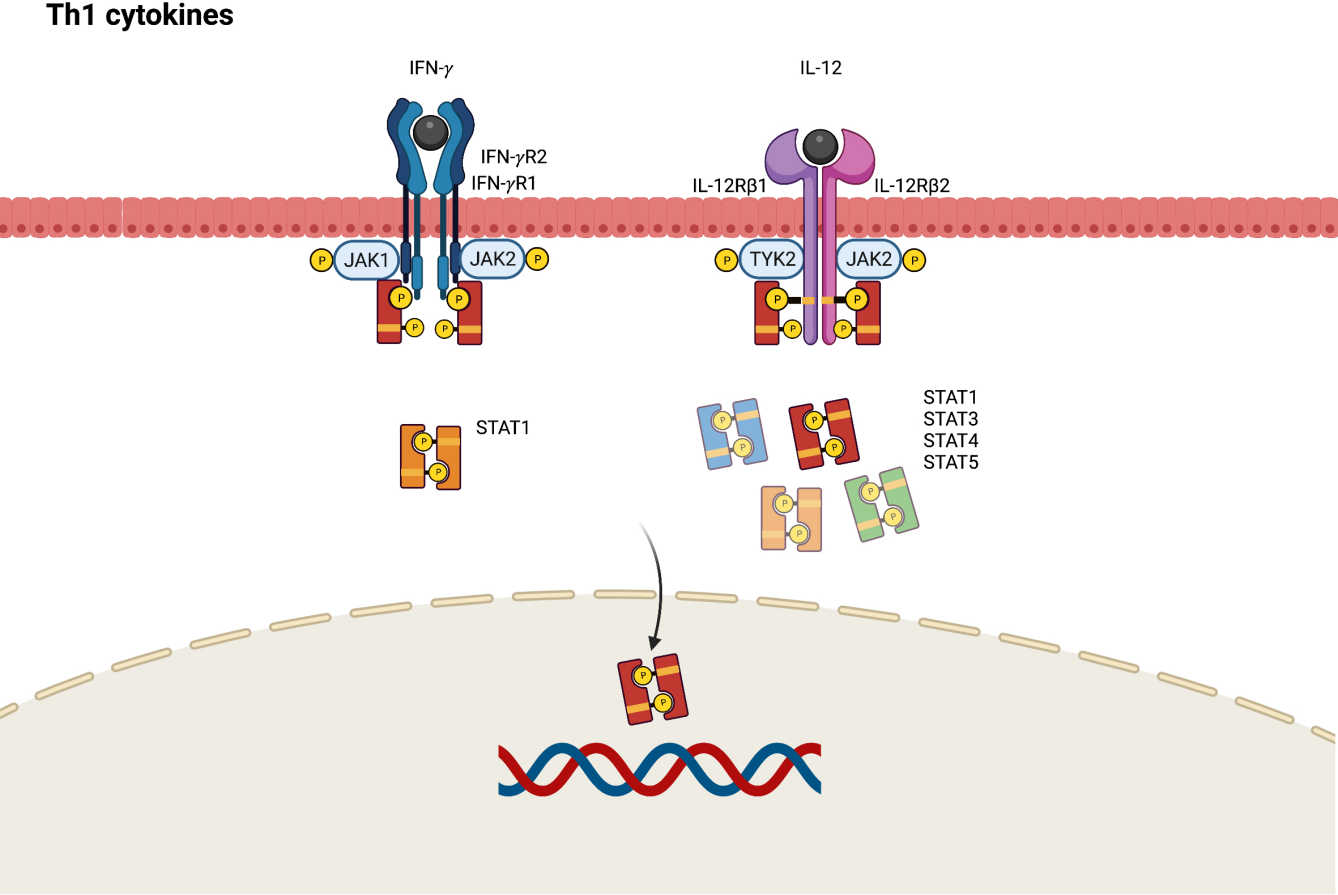
JAK-STAT signaling pathway in Th1 axis immune response of atopic dermatitis. IFN, interferon; IL, interleukin; JAK, Janus kinase; R, receptor; STAT, signal transducer and activator of transcription.

Although Th1 cells are more predominant in the pathogenesis of psoriasis, Th1 skewed immune response also plays a role in the chronic phase of AD. Higher levels of Th1 cytokines including interferon-gamma (IFN- γ), IL-12, and granulocyte colony stimulating factor (G-CSF) are found in chronic AD lesions (61, 62). In the pathogenesis of AD, IL-12 secreted by dendritic cells can polarize Th1 cells from naïve T-helper cells and inhibit the activation of Th2 cells. The IL-12 signaling pathway is initiated by binding to a distinct heterodimeric receptor comprising interleukin 12 receptor beta (IL-12Rβ)1 and IL-12Rβ2 subunits (63). Subsequently, JAK2 and TYK2 are polarized, resulting in the mediation of signal transduction through the activation of STAT4, and to a lesser degree STAT1, STAT3, and STAT5 (**Figure 4**) (64).

IFN-γ contributes to epidermal dysfunction by reducing the expression levels of ceramides and long-chain fatty acids (65). JAK1 and JAK2 are activated by the conjugation of IFN-γ and its receptor, which comprises IFN-gamma receptor (IFN-γR)1 and IFN-γR2. This phosphorylates STAT1 to transduce transcription signals into the nucleus and bind to the γ-IFN-activated sequence, affecting IFN-γ-induced gene expression (66).

#### 2.3.4 Skin barrier dysfunction

JAK1 is involved in the pathogenesis of AD through the signaling pathways of IL-4, IL-5, IL-13, IL-22, TSLP, and IFN-γ. Hyperactivation of JAK1 results in the overexpression of cutaneous serine protease, causing skin barrier dysfunction (67). Furthermore, the STAT3 signal is a key transcriptional factor that modulates keratinocyte differentiation and maintains skin integrity. In a murine dry skin model, delgocitinib, a JAK inhibitor blocking JAK1, JAK2, and JAK3, inhibited the activation of STAT3 and improved skin barrier functionality by increasing epidermis-associated proteins such as FLG, loricrin, and natural moisturizing factors (68).

#### 2.3.5 Pruritus and pain in AD

The etiology of pruritus in AD involves the interplay between hyperinnervation of the epidermis; increased pruritogens such as histamine, 5 hydroxytryptamine, nerve growth factor (NGF), and substance P; TSLP; and IL-31 (69, 70). The sensation of itchiness is transmitted by cutaneous unmyelinated C-fiber and myelinated Aδ fiber afferents originating from the dorsal root ganglion. The activation of STAT3 mediates astrogliosis in the spinal dorsal horn, acting as an amplifier of itchiness and leading to chronic pruritus (71). In this signaling pathway, lipocalin-2 (LCN2) was identified as the distinct upregulated factor that enhances pruritus signals and triggers the vicious itch-scratch cycle (72).

Notably, IL-31 was observed to stimulate nerve elongation and directly communicate with the primary cutaneous afferent neurons required by pruritogens to transduce itch signals (73, 74). In an AD-like murine model, topical delgocitinib was implicated to suppress IL-31-promoted nerve elongation *in vivo* and morphological changes of peripheral nerves from the dorsal root ganglion *in vitro* (75). The topical application of delgocitinib also resulted in substantially reduced scratching behavior in AD mouse models.

Patients with AD may also experience skin pain, demonstrating that the AD symptom burden extends beyond itchiness. The pain inflicted by AD can cause sleep disturbances and decrease quality of life. Evidence supports that the underlying mechanism for the pain response in AD is inflammation induced by proinflammatory cytokines and neuropathic pain of the peripheral nervous system (76). Cytokines involved in the JAK–STAT pathway also contribute to pain modulation. GM-CSF can induce hyperalgesia (77), whereas IL-17 may result in allodynia (78). TNF-α in the spinal cord is believed to modulate neuropathic pain sensations and sensitize pain-receptors for both mechanical and thermal hyperalgesia (79). Hence, patients receiving JAK inhibitors such as baricitinib reported reduced skin pain according to a numerical rating scale (NRS) (80).

## 3 JAK inhibitors for AD

Oral and topical JAK inhibitors are novel small molecules developed for treating various autoimmune and inflammatory diseases. Although few JAK inhibitors have been approved by the U.S. Food and Drug Administration (FDA) for the treatment of dermatological diseases, emerging evidence demonstrated the therapeutic efficacy of JAK inhibitors in treating numerous skin diseases, including AD, alopecia areata, psoriasis, and vitiligo (81). The selective inhibition of various JAK inhibitors provides a diverse array of clinical applications through the modulation of various cytokine signaling pathways.

### 3.1 Topical JAK inhibitors for AD

#### 3.1.1 Tofacitinib

Tofacitinib is a first-generation small molecules designed to selectively block JAK1 and JAK3, and to a lesser extent, JAK2 and TYK2. Tofacitinib has oral and topical formulations, but only the oral form of tofacitinib is commercially available as of writing this paper. Oral tofacitinib was approved by the U.S. FDA for the treatment of rheumatoid arthritis, psoriatic arthritis, ankylosing spondylitis, polyarticular juvenile idiopathic arthritis, and ulcerative colitis (82). Although the oral administration of tofacitinib has demonstrated clinical efficacy in the treatment of recalcitrant AD without significant AEs (83, 84), clinical application remains limited because of the paucity of randomized controlled trials (RCTs). Nevertheless, the relevant literature has reported that topical tofacitinib therapy for mild-to-moderate AD resulted in a significantly greater mean percentage change in Eczema Area and Severity Index (EASI) score, Physician’s Global Assessment (PGA) response, and body surface area than the vehicle treatment (**Table 2**) (85). Notably, topical tofacitinib demonstrated rapid efficacy during the first week of treatment and continuous improvement throughout a 4-week treatment course. The patients were reported to be well-tolerated to topical tofacitinib without any serious adverse events nor death (85).

**Table 2:** Topical JAK inhibitors for atopic dermatitis.

JAK inhibitors	FDA approval for AD	RCT	Inclusion	Treatment	Treatment period	Patients (n)	Sex (M/F)	Age	Baseline EASI score	Primary endpoint	Results
Tofacitinib (JAK1/3)	X	Phase 2a RCT (NCT02001181) (85)	Adults with mild-to-moderate AD	2% tofacitinib BID Vehicle BID	4 weeks	35 34	16/19 6/18	32.4 (9.8) 30.4 (10.4)	5.4 (2.6) 5.7 (3.1)	%EASI at Week 4	-81.7 (6.3)% -29.9 (6.5)%
Ruxolitinib (JAK1/2)	V	Phase 2 RCT (NCT03011892) (86)	Adults with mild-to-moderate AD	1.5% Ruxolitinib BID 1.5% Ruxolitinib QD 0.5% Ruxolitinib QD 0.15% Ruxolitinib QD 0.1% TAC BID* Vehicle BID	12 weeks (8 weeks RCT+4 weeks OLE)	50 52 51 51 51 52	26/24 21/31 24/27 25/26 23/28 20/32	39.0 (15.7) 39.1 (14.5) 38.6 (13.7) 38.4 (13.8) 38.5 (16.2) 34.8 (15.0)	8.4 (4.7) 8.4 (4.7) 8.5 (4.8) 8.2 (4.5) 8.4 (4.7) 8.6 (5.1)	%EASI at Week 4	-71.6 (6.4)% -66.7 (6.4)% -52.8 (6.5)% -44.9 (6.5)% -59.5 (6.5)% -11.9 (6.6)%
		Phase 3 RCT (TRuE-AD1, NCT03745638) (87)	Age≧12 with AD	1.5% Roxolitinib BID 0.75% Ruxolitinib BID Vehicle BID	52 weeks (8 weeks RCT+44 weeks OLE)	253 252 126	95/158 98/154 47/79	33.7 (17.2) 36.8 (19.1) 35.2 (18.1)	7.9 (4.6) 8.2 (4.8) 7.4 (4.3)	IGA treatment success at week 8§	53.8 (3.1)% 50.0 (3.2)% 15.1 (3.2)%
		Phase 3 RCT (TRuE-AD2, NCT03745651) (87)	Age≧12 with AD	1.5% Roxolitinib BID 0.75% Ruxolitinib BID Vehicle BID	52 weeks (8 weeks RCT+44 weeks OLE)	246 248 124	96/150 98/150 44/80	35.9 (18.0) 35.8 (18.5) 38.9 (18.9)	7.8 (4.9) 8.1 (5.0) 8.2 (5.2)	IGA treatment success at week 8§	51.3 (3.3)% 39.0 (3.2)% 7.6 (2.4)%
Delgocitinib/ JTE052 (JAK1/2/3, TYK2)	X	Phase 2 RCT (JapicCTI -152887) (88)	Age≧16 with AD	3% Delgocitinib BID 1% Delgocitinib BID 0.5% Delgocitinib BID 0.25% Delgocitinib BID 0.1% Tacrolimus BID Vehicle BID	4 weeks	65 66 65 69 30 31	42/23 48/18 39/26 48/21 14/16 19/12	32.3 (10.6) 28.6 (8.7) 29.5 (9.2) 31.5 (10.5) 33.1 (11.6) 31.6 (9.6)	14.9 (3.8) 16.2 (4.3) 15.0 (4.4) 15.6 (4.3) 14.8 (4.0) 15.3 (4.9) ¶	%mEASI at Week 4	-72.9% -54.9% -57.1% -41.7% NA -12.2%
		Phase 2 RCT (JapicCTI-173553) (89)	Age of 2-15 with AD	0.5% Delgocitinib BID 0.25% Delgocitinib BID Vehicle BID	4 weeks	34 34 35	18/16 22/12 18/17	8.5 (4.4) 8.4 (3.8) 8.6 (4.0)	11.1 (4.6) 10.5 (4.2) 11.3 (4.7)¶	%mEASI at Week 4	-61.8% -54.2% -4.8%
		Phase 3 RCT (JapicCTI-173554) (90)	Age≧16 with AD	0.5% Delgocitinib BID Vehicle BID	28 weeks (4 weeks RCT+24 weeks OLE)	106 52	64/42 34/18	31.4 (9.6) 32.3 (11.2)	14.2 (3.5) 14.5 (3.8)¶	%mEASI at Week 4	-44.4% 1.7%
		Phase 3 RCT (JapicCTI-184064) (91)	Age of 2-15 with AD	0.25% Delgocitinib BID Vehicle BID	28 weeks (4 weeks RCT+24 weeks OLE)	69 68	39/30 31/37	8.2 (3.9) 8.3 (3.7)	10.7 (4.3) 10.6 (4.2)	%mEASI at Week 4	-39.3% 10.9%
		Phase 3 OL (JapicCTI‐173555) (91)	Age≧16 with AD	0.5% Delgocitinib BID	52 weeks	506	318/188	32.6 (10.8)	10.5 (5.6)¶	Adverse events	69.0% had adverse events.
Brepocitinib/ PF-06700841 (JAK1, TYK2)	X	Phase 2 RCT (NCT03903822) (92)	Age 12-75 with mild-to-moderate AD	1.0% PF-06700841 BID 0.3% PF-06700841 BID Vehicle BID 3.0% PF-06700841 QD 1.0% PF-06700841 QD 0.3% PF-06700841 QD 0.1% PF-06700841 QD Vehicle QD	6 weeks	37 36 36 36 37 36 37 37	17/20 20/16 17/19 21/15 14/23 12/24 18/19 17/20	38.1 (15.3) 39.4 (17.3) 42.3 (18.2) 40.5 (12.3) 38.4 (12.9) 43.4 (16.4) 40.8 (15.4) 39.1 (16.8)	NA	%EASI at Week 6	-75.0% -58.6% -47.6% -67.9% -70.1% -64.6% -58.3% -44.4%
Ifidancitinib/ ATI-502 (JAK1/3)	X	Phase 2 OL (NCT03585296) (93)	Adults with AD	AT-502 QD	4 weeks	22	9/13	NA	NA	Number of subjects with TEAEs	NA

^∫^Age, baseline EASI score, and results are presented as mean (SD) or least-squares mean only.

*0.1% TAC BID for the first 4 weeks, then vehicle thereafter.

^§^Investigator’s Global Assessment score of 0/1 and ≥2-grade improvement from baseline

^¶^Modified EASI score

%EASI, mean percentage change of Eczema Area and Severity Index score from baseline; % mEASI, least-squares mean percentage change of modified Eczema Area and Severity Index score from baseline; AD, atopic dermatitis; BID, twice daily; EASI, Eczema Area and Severity Index; FDA, Food and Drug Administration; IGA, Investigator’s Global Assessment; JAK, Janus kinase; QD, once daily; mEASI, modified Eczema Area and Severity Index; OL, open-label study; OLE, open-label extension; RCT, randomized controlled trial; TAC, triamcinolone acetonide cream; TEAE, treatment-emergent adverse event. NA, Not Available.

#### 3.1.2 Ruxolitinib

Ruxolitinib is a selective JAK1 and JAK2 inhibitor. Its oral formulation has been approved by the U.S. FDA for the treatment of myelofibrosis, polycythemia vera, and graft-versus-host disease (94). Topical 1.5% ruxolitinib cream has been approved by the U.S. FDA for the short-term and noncontinuous chronic treatment of mild-to-moderate AD in patients aged ≥12 years (95). Compared with the vehicle, ruxolitinib therapy resulted in significant improvement of EASI score and Investigator’s Global Assessment (IGA) scores among patients with AD (86). Notably, the rapid activation of ruxolitinib accounted for a significant reduction in itching within the first 12 hours after application, with sustainable efficacy throughout an 8-week treatment period (87). No differences in application site reactions were observed between ruxolitinib cream and the vehicle, indicating the favorable skin tolerability of ruxolitinib cream (87).

#### 3.1.3 Delgocitinib (JTE-052)

As a pan-JAK inhibitor, topical delgocitinib (Corectim) has been approved by Japan Tobacco for the treatment of AD; however, this drug has yet to be approved by the U.S. FDA (96). Patients aged 2–15 and ≥16 years who were treated with 0.25% and 0.5% formulations of topical delgocitinib, respectively, had substantially higher modified EASI (mEASI) scores than the vehicle group. The majority of AEs were mild and well tolerated, such as nasopharyngitis, Kaposi varicelliform eruption, and acne (89–91). Application site reactions were rare and mostly occurred within the first 2 weeks of treatment, indicating high skin tolerability of topical delgocitinib (97). Furthermore, blood tests revealed no plasma concentration of delgocitinib in most patients, indicating minimal systemic absorption of delgocitinib (91).

#### 3.1.4 Brepocitinib (PF-06700841)

Brepocitinib (PF-06700841) is a selective JAK1 and TYK2 inhibitor. Oral brepocitinib for the treatment of plaque psoriasis, alopecia areata, inflammatory bowel disease, and other inflammatory diseases remains under investigation (98–100). TYK2 is phosphorylated in the IL-4, IL-12, IL-13, and IL-22 cytokine signaling pathways; thus, brepocitinib is believed to be a potentially effective treatment for AD. The efficacy of topical brepocitinib in treating patients between the ages of 12–75 years with AD was evaluated by a dose-ranging, phase 2b RCT (92). In the RCT, brepocitinib met the primary endpoint of a mean change in EASI score at Week 6 and demonstrated superiority over the vehicle treatment. The rate of AEs in the brepocitinib group was generally lower than that in the vehicle group.

#### 3.1.5 Ifidancitinib (ATI-502)

ATI-502, a topical JAK1 and JAK3 inhibitor, was investigated for efficacy and safety in the treatment of AD in a phase 2 open-label study (93). ATI-502 demonstrated high efficacy through improved EASI score, IGA score, and itch-NRS. ATI-502 was also demonstrated to be well tolerated, with primary safety endpoints met (93, 101).

### 3.2 Oral JAK inhibitors for AD

#### 3.2.1 Upadacitinib (ABT-494)

Upadacitinib, an oral reversible JAK inhibitor with greater inhibitory potency for JAK1 than JAK2, JAK3, and TYK2, has been approved by the U.S. FDA for treating AD, rheumatoid arthritis, and psoriatic arthritis (102). According to the FDA recommendation, the strategy for upadacitinib treatment in patients with AD aged 12-64 years is initiation from 15 mg/day and titration to 30 mg/day if necessary (**Table 3**). The same guideline proposed a 15 mg/day dose for patients aged ≥65 years (102). In two consecutive phase 3 RCTs (Measure Up 1 and Measure Up 2), both the 30 and 15 mg doses of upadacitinib presented significantly higher EASI and IGA responses than the placebo (104). Furthermore, upadacitinib combined with TCS demonstrated high tolerability and sustainable efficacy over a 52-week treatment period (123).

**Table 3:** Oral JAK inhibitors for atopic dermatitis.

JAK inhibitors	FDA approval for AD	RCT	Inclusion	Treatment	Treatment period	Patients (n)	Sex (M/F)	Age	Baseline EASI score	Primary endpoint	Results
Upadacitinib (JAK1)	V	Phase 2b RCT (NCT02925117) (103)	Adults with moderate-to-severe AD	Upadacitinib 30mg QD Upadacitinib 15mg QD Upadacitinib 7.5mg QD Placebo	88 weeks (16 weeks treatment+72 weeks withdraw)	42 42 42 41	22/20 30/12 28/14 24/17	39.9 (15.3) 38.5 (15.2) 41.5 (15.4) 39.9 (17.5)	28.2 (11.6) 31.4 (12.3) 31.4 (15.8) 32.6 (14.5)	%EASI at Week 16	-74 (6.1)% -62 (6.1)% -39 (6.2)% -23 (6.4)%
		Phase 3 RCT (Measure Up 1, NCT03569293) (104)	Adults with moderate-to-severe AD	Upadacitinib 30mg QD Upadacitinib 15mg QD Placebo	16 weeks	285 281 281	155/130 157/124 144/137	33.6 (NA) 34.1 (NA) 34.4 (NA)	29.0 (11.1) 30.6 (12.8) 28.8 (12.6)	EASI75 at Week 16	227 (79.7%) 196 (69.6%) 46 (16.3%)
		Phase 3 RCT (Measure Up 2, NCT03607422) (104)	Adults with moderate-to-severe AD	Upadacitinib 30mg QD Upadacitinib 15mg QD Placebo	16 weeks	282 276 278	162/120 155/121 154/124	34.1 (NA) 33.3 (NA) 33.4 (NA)	29.7 (12.2) 28.6 (11.7) 29.1 (12.1)	EASI75 at Week 16	206 (72.9%) 166 (60.1%) 37 (13.3%)
		Phase 3 RCT (AD Up, NCT03568318) (105)	Age of 12-75 with AD	Upadacitinib 30mg+TCS Upadacitinib 15mg QD+TCS Placebo+TCS	16 weeks treatment+260 weeks BE	297 300 304	190/107 179/121 178/126	35.5 (NA) 32.5 (NA) 34.3 (NA)	29.7 (11.8) 29.2 (11.8) 30.3 (13.0)	EASI75 at Week 16	229 (77.1%) 194 (64.6%) 80 (26.4%)
		Phase 3b RCT (Heads Up, NCT03738397) (106)	Adults with moderate-to-severe AD	Upadacitinib 30mg QD Dupilumab∫	24 weeks	348 344	183/165 194/150	36.6 (14.6) 36.9 (14.1)	30.8 (12.5) 28.8 (11.5)	EASI75 at Week 16	247 (71.0%) 210 (61.1%)
Baricitinib/ LY3009104 (JAK1/2)	X	Phase 2b RCT (NCT02576938) (107)	Adults with moderate-to-severe AD	Baricitinib 4mg QD+TCS Baricitinib 2mg QD+TCS Placebo+TCS	16 weeks	38 37 49	22/16 22/15 35/14	32.5 42.0 35.0*	19.5 22.1 22.1*	EASI50 at Week 16	23 (61%) 21 (57%) 18 (37%)
		Phase 3 RCT (BREEZE-AD1, NCT03334396) (80)	Adults with moderate-to-severe AD	Baricitinib 4mg QD Baricitinib 2mg QD Baricitinib 1mg QD Placebo	16 weeks	125 123 127 249	83/4/2 82/41 78/49 148/101	37 (12.9) 35 (13.7) 36 (12.4) 35 (12.6)	32 (12.7) 31 (11.7) 29 (11.8) 32 (13.0)	vIGA-AD 0/1 at Week 16	21 (16.8%) 14 (11.4%) 15 (11.8%) 12 (4.8%)
		Phase 3 RCT (BREEZE-AD2, NCT03334422) (80)	Adults with moderate-to-severe AD	Baricitinib 4mg QD Baricitinib 2mg QD Baricitinib 1mg QD Placebo	16 weeks	123 123 125 244	82/41 65/58 80/45 154/90	34 (14.1) 36 (13.2) 33 (10.0) 35 (13.0)	33 (12.7) 35 (16.0) 33 (12.7) 33 (12.8)	vIGA-AD 0/1 at Week 16	17 (13.8%) 13 (10.6%) 11 (8.8%) 11 (4.5%)
		Phase 3 LTE (BREEZE-AD3, NCT03334435) (108)	Adults with moderate-to-severe AD	Baricitinib 4mg QD Baricitinib 2mg QD	68 weeks	70 54	42/28 28/26	36.7 (15.5) 32.8 (12.7)	28.1 (10.6) 24.9 (8.7)	vIGA-AD 0/1 at Week 68	33 (47.1%) 32 (59.3%)
		Phase 3 RCT (BREEZE-AD4, NCT03428100) (109)	Adults with moderate-to-severe AD	Baricitinib 4mg QD+TCS Baricitinib 2mg QD+TCS Baricitinib 1mg QD+TCS Placebo+TCS	16 weeks	92 185 93 93	57/35 133/52 58/35 49/44	38.7 (13.3) 37.3 (13.6) 38.9 (14.0) 38.7 (13.6)	NA	EASI75 at Week 16	29 (31.5%0 51 (27.6%) 21 (22.6%) 16 (17.2%)
		Phase 3 RCT (BREEZE-AD5, NCT03435081) (110)	Adults with moderate-to-severe AD	Baricitinib 2mg QD Baricitinib 1mg QD Placebo	16 weeks	146 147 147	69/77 75/72 80/67	40 (15) 40 (17) 39 (17)	26.6 (11) 27.7 (12) 27.0 (11)	EASI75 at Week 16	44 (30%) 19 (13%) 12 (8%)
		Phase 3 OLLTE (BREEZE-AD6, NCT03559270) (111)	Adults with moderate-to-severe AD	Ongoing	16 weeks	NA	NA	NA	NA	EASI75 at Week 16	NA
		Phase 3 RCT (BREEZE-AD7, NCT03733301) (112)	Adults with moderate-to-severe AD	Baricitinib 4mg QD+TCS Baricitinib 2mg QD+TCS Placebo+TCS	16 weeks	111 109 109	75/36 70/39 71/38	33.9 (11.4) 33.8 (12.8) 33.7 (13.2)	30.9 (12.6) 29.3 (11.9) 28.5 (12.3)	vIGA-AD 0/1 at Week 16	34 (31%) 26 (24%) 16 (15%)
Abrocitinib/ PF-04965842 (JAK1)	V	Phase 2 RCT (NCT02780167) (113)	Adults with moderate-to-severe AD	Abrocitinib 200mg QD Abrocitinib 100mg QD Abrocitinib 30mg QD Abrocitinib 10mg QD Placebo+TM	12 weeks	55 56 51 49 56	28/27 31/25 22/29 21/28 21/35	38.7 (17.6) 41.1 (15.6) 37.6 (15.9) 44.3 (15.9) 42.6 (15.1)	24.6 (13.5) 26.7 (11.8) 22.1 (10.7) 28.1 (13.1) 25.4 (12.9)	vIGA 0/1 at Week 12	21 (43.8%) 16 (29.6%) 4 (8.9%) 5 (10.9%) 3 (5.8%)
		Phase 3 RCT (JADE MONO-1,NCT03349060) (114)	Age≧12 with moderate-to-severe AD	Abrocitinib 200mg QD Abrocitinib 100mg QD Placebo	12 weeks	154 156 77	81/73 90/66 49/28	33.0 (17.4) 32.6 (15.4) 31.5 (14.4)	30.6 (14.1) 31.3 (13.6) 28.7 (12.5)	vIGA 0/1 at Week 12 / EASI75 at Week 12	67 (44%) 37 (24%) 6 (8%) 96 (63%) 62 (40%) 9 (12%)
		Phase 3 RCT (JADE MONO-2,NCT03575871) (115)	Age≧12 with moderate-to-severe AD	Abrocitinib 200mg QD Abrocitinib 100mg QD Placebo	12 weeks	155 158 78	88/67 94/64 47/31	33.5 (14.7) 37.4 (15.8) 33.4 (13.8)	29.0 (12.4) 28.4 (11.2) 28.0 (10.2)	vIGA 0/1 at Week 12 / EASI75 at Week 12	55 (38.1%) 44 (28.4%) 7 (9.1%) / 94 (61.0%) 69 (44.5%) 8 (10.4%)
		Phase 3 RCT (JADE TEEN, NCT03796676) (116)	Age of 12-17 with moderate-to-severe AD	Abrocitinib 200mg QD+TM Abrocitinib 100mg QD+TM Placebo+TM	12 weeks	94 95 96	56/38 45/50 44/52	15.0 16.0 14.0*	29.5 (12.2) 31.0 (12.8) 29.2 (12.7)	vIGA 0/1 at Week 12 / EASI75 at Week 12	43 (46.2%) 37 (41.6%) 23 (24.5%) / 67 (72.0%) 61 (68.5%) 39 (41.5%)
		Phase 3 RCT (JADE COMPARE, NCT03720470) (117)	Adults with moderate-to-severe AD	Abrocitinib 200mg QD+TM Abrocitinib 100mg QD+TM Dupilumab^∫^+TM Placebo	12 weeks	238 226 242 131	120/118 104/122 108/134 77/54	37.3 (14.8) 38.8 (14.5) 37.1 (14.6) 37.4 (15.2)	30.3 (13.5) 32.1 (13.1) 30.4 (12.0) 31.0 (12.6)	vIGA 0/1 at Week 12 / EASI75 at Week 12	106 (48.4%) 86 (36.6%) 88 (36.5%) 18 (14.0%) / 154 (70.3%) 138 (58.7%) 140 (58.1%) 35 (27.1%)
		Phase 3 RCT (JADE REGIMEN, NCT03627767) (118)	Age≧12 with moderate-to-severe AD	Abrocitinib 200mg QD Abrocitinib 100mg QD Placebo	54 weeks (12 weeks OLi, 30 weeks treatment, 12 weeks OLr)	266 265 267	150/116 148/117 141/126	28.0 29.0 29.0*	27.2 27.7 26.9*	the rate of flare-up§	44 (16.5%) 105 (39.6%) 207 (77.5%)
		Phase 3 LTE (JADE EXTEND, NCT03422822) (119)	Adults with moderate-to-severe AD	Abrocitinib 200mg QD Abrocitinib 100mg QD	12 weeks	73 130	35/38 61/69	37.3 (14.5) 38.6 (14.8)	31.2 (12.4) 29.6 (11.2)	vIGA 0/1 at Week 12	Prior dupilumab responder 25 (83.3%) 30 (76.9%) / Nonresponder 17 (47.2%) 25 (35.2%)
		Phase 3 RCT (JADE DARE, NCT04345367) (120)	Adults with moderate-to-severe AD	Abrocitinib 200mg QD Dupilumab^∫^	26 weeks	362 365	193/169 204/161	34.6 (14.6) 35.5 (13.3)	28.1 (11.5) 28.1 (11.9)	PP-NRS4 at week 2 / EASI-90 at week 4	172 (48%) 93 (26%) / 101 (29%) 53 (15%)
Gusacitinib/ASN002 (JAKs, SYK)	X	Phase 1b RCT (NCT03139981)	Adults with moderate-to-severe AD	ASN002 80mg QD ASN002 40mg QD ASN002 20mg QD Placebo	4 weeks	9 9 9 9	5/4 5/4 5/4 3/6	33.1 (10.4) 42.4 (13.9) 38.2 (14.4) 29.9 (9.3)	28.2 (11.7) 21.8 (6.2) 29.0 (13.5) 21.6 (6.2)	TEAEs	Headache, nausea, diarrhea
		Phase 2b RCT (NCT03531957) (121)	Adults with moderate-to-severe AD	ASN002 80mg QD ASN002 40mg QD ASN002 20mg QD Placebo	36 weeks (12 weeks treatment+24 weeks OLE)	NA	NA	NA	NA	cEASI at Week 12	NA
Ivarmacitinib/ SHR0302 (JAK1)	X	Phase 2 RCT (NCT04162899) (122)	Adults with moderate-to-severe AD	SHR0302 8mg QD SHR0302 4mg QD Placebo	12 weeks	35 35 35	23/12 20/15 26/9	35.2 (14.8) 38.5 (14.8) 30.3 (12.6)	25.4 (11.3) 30.5 (15.7) 28.2 (12.1)	vIGA 0/1 at Week 12	19 (54.3%) 9 (25.7%) 2 (5.7%)

Data are n, n (%), mean, mean (SD), or median*.

^∫^ 300 mg dupilumab was injected subcutaneously every 2 weeks after a loading dose of 600 mg.

^§^ Defined as ≥50% reduction in initial EASI response at week 12 with a new IGA score of ≥2

^❡^ AD, atopic dermatitis; BE, blinded extension; cEASI, change of EASI score from baseline; EASI50/75, ≥50/75% improvement of EASI score from baseline; JAK, Janus kinase; LOR, loss of response; LTE, long-term extension; OL, open-label; OLi, open-label induction period; OLr, open-label rescue period; PP-NRS4, 4 point or more improvement in Peak Pruritus Numerical Rating Scale; SYK, spleen tyrosine kinase; TCS, topical corticosteroid; TM, topical medication; vIGA-AD 0/1, validated Investigator’s Global Assessment of AD score of 0 or 1 with a 2-point or greater improvement from baseline. NA, Not Available.

Dupilumab was designed as a fully human monoclonal antibody targeting IL-4 and IL-13 receptors and was considered a promising biologics for AD. Nevertheless, less than 50% of patients achieved clear or almost clear skin during a 16-week course of dupilumab monotherapy (13), leading to the search for more effective therapeutic options. Compared with the 50% success rate for dupilumab, patients treated with upadacitinib presented substantially better results, with ≥75% of patients achieving an improvement in EASI score from baseline (EASI75) (106). Upadacitinib was also superior to dupilumab in terms of several secondary endpoints, including the percentage of patients achieving ≥90% and 100% improvement in EASI score (EASI90 and EASI100) and the percentage change of Worst Pruritus NRS at week 16 (106). In contrast, patients receiving upadacitinib presented higher rates of serious infection, eczema herpeticum, herpes zoster, and laboratory-related AEs than patients receiving dupilumab (106).

Overall, the rate of AEs associated with upadacitinib was relatively low, and the frequency of AEs was reported to be similar between the 15 and 30 mg doses. The 30 mg dose was associated with a slightly higher rate of treatment discontinuation because of AEs and serious infection than the 15 mg dose, which was considered to be attributed to the dosage effect (124). The most common treatment-emergent AEs (TEAEs) of upadacitinib were acne, upper respiratory tract infection, nasopharyngitis, and headache (104). Nevertheless, long-term use of upadacitinib demonstrated a favorable risk–benefit profile in treating patients with moderate-to-severe AD (124). Therefore, upadacitinib remains a promising orally systemic therapeutic alternative to conventional therapies and subcutaneous biologics for patients with moderate-to-severe AD.

#### 3.2.2 Baricitinib (LY3009104)

Baricitinib was approved by the U.S. FDA as a selective JAK1 and JAK2 inhibitor for the indications of rheumatoid arthritis and Coronavirus Disease 2019 (COVID-19) but not yet for atopic dermatitis (125). Nevertheless, it has been approved in European union and Japan for adult patients with moderate to severe AD (126). Dosages of 4, 2, and 1 mg baricitinib were used in various RTCs to evaluate efficacy. In a phase 2 study, baricitinib 4 and 2 mg both effectively improved EASI scores when combined with TCS treatment (107). Among the patients with moderate-to-severe AD who did not respond to TCS therapy, monotherapy of baricitinib provided rapid itch reduction and demonstrated significant amelioration of AD severity relative to the placebo. Additionally, baricitinib improved the patients’ quality of life with fair tolerability (80). Furthermore, baricitinib demonstrated sustained long-term efficacy of up to 68 weeks and greater amelioration of itch-related symptoms throughout long-term administration (108). The frequency of serious infections and opportunistic infections was similar to that of the placebo (111). Notably, the 4-mg dose of baricitinib presented a higher risk of herpes simplex than the 2-mg dose, which was considered to be attributed to the dosage effect (111). Overall, baricitinib alone or in combination with TCS is an effective and well-tolerated systemic therapy for patients with moderate-to-severe AD.

#### 3.2.3 Abrocitinib (PF-04965842)

Abrocitinib, a selective JAK1 inhibitor, is U.S. FDA approved for treating refractory, moderate-to-severe AD, with a recommended dosage of 100 mg/day (127). A dose-ranging phase 2b study reported that only abrocitinib 200 and 100 mg/day demonstrated significant efficacy and tolerability compared with the placebo, whereas the outcomes for the 30 and 10 mg doses of abrocitinib were statistically nonsignificant (113). Several phase 3 RCTs have demonstrated that the 200 and 100 mg doses of abrocitinib as monotherapy or concomitant with topical agents were highly beneficial treatments for patients aged ≥12 with moderate-to-severe AD (113–116). Compared with dupilumab, the 200 mg dose of abrocitinib showed a better IGA 0/1 or EASI75 response during the 16-week treatment period but did not reach statistical significance (117). The 200-mg dose of abrocitinib was superior to dupilumab with respect to itch response at week 2 (117). In a recent published phase 3 trial, abrocitinib 200 mg once per day provided higher rates of early itch reduction than dupilumab 300 mg (a 4 point or higher improvement in Peak Pruritus Numerical Rating Scale [PP-NRS4] response at week 2) which complements the above findings (120). Notably, a statistical superiority of abrocitinib versus dupilumab on the primary endpoints with faster onset of high-level improvement of disease signs (EASI-90 response at week 4), and the difference in EASI-90 response remained significant at week 16 (120). Furthermore, abrocitinib demonstrated sustainable long-term efficacy through 52 weeks (118). The results of these trials are supporting the potential important role of JAK1 inhibition in patients who need fast relief of pruritus and skin inflammation in atopic dermatitis (113–118, 120).

The most common TEAEs associated with abrocitinib were nausea, upper respiratory tract infection, nasopharyngitis, and headache, which were similar to the AEs reported for other JAK inhibitors (114). Nausea, the most frequent gastrointestinal AE, occurred only in patients with fasted status, indicating that the gastrointestinal symptoms may be caused by the local gastric concentration of abrocitinib (128). Accordingly, abrocitinib is an effective and safe small molecules for treating patients aged ≥12 years with moderate-to-severe AD.

#### 3.2.4 Gusacitinib (ASN002)

ASN002 is a potent dual inhibitor, which simultaneously blocks both spleen tyrosine kinase (SYK) and JAKs, but is yet to receive U.S. FDA approval. SYK is a nonreceptor tyrosine kinase involved in the regulation of B cells, the differentiation of dendritic cells, the inhibition of keratinocyte differentiation, and Th17 signaling (129).

Patients with AD who were treated with ASN002 exhibited robust tissue improvement in cellular infiltration (AD lesions), inflammatory pathogenesis, and skin barrier dysfunction (129). Clinically, ASN002 is a safe and well-tolerated therapy for patients with moderate-to-severe AD. Mild AEs were observed, including headache, nausea, and diarrhea. However, only the highest dose (80 mg, as opposed to 40 or 20 mg) of ASN002 demonstrated significantly greater amelioration of pruritus than the placebo. A phase 2b RCT was completed but the data were unavailable at the time of publication.

#### 3.2.5 Ivarmacitinib (SHR0302)

SHR0302 is a highly selective JAK1 inhibitor that also exercises a mild JAK2 inhibiting effect. In laboratory studies, SHR0302 was revealed to minimize the risk of AEs such as neutropenia and anemia (36). SHR0302 is easily absorbed through oral administration, and pharmacodynamic evidence supports a once-daily treatment strategy. A phase 2 study demonstrated that both 8 and 4 mg doses of SHR0302 were superior to a placebo in achieving IGA0/1, EASI75, and an improvement of ≥3 points on the NRS (NRS-3) (122). The oral therapy was also well tolerated, without clinically significant changes in hematology parameters. Further studies evaluating the efficacy and safety of SHR0302 are warranted.

### 3.3 Evidence of comparative effectiveness from network meta-analysis

Numerous novel small molecules and biologics have been developed. Choosing the most appropriate treatment for patients with AD recalcitrant to conventional treatment can be challenging. Among all steroid-sparing topical treatments for AD, twice-daily doses of tofacitinib 2%, delgocitinib 3%, and ruxolitinib 1.5% demonstrated superior efficacy when compared with other topical JAK and PDE4 inhibitors (130). Regarding the choice of systemic therapy, Drucker et al. conducted a network meta-analysis to compare the efficacy among all systemic immunomodulatory therapies for AD (131). In the reported analysis, the 200-mg dose of abrocitinib and 30-mg dose of upadacitinib demonstrated greater improvements in EASI scores than dupilumab, whereas the 15-mg dose of upadacitinib demonstrated similar efficacy to dupilumab.

### 3.4 Safety for JAK inhibitors in AD

In addition to the efficacy of these small molecules, drug safety is also a priority for clinicians to make the most appropriate decision in treating AD. The most commonly reported adverse events associated with JAK inhibitors are generally not serious, such as upper respiratory infections, urinary tract infections, and nasopharyngitis, hematological abnormalities, nausea, and headache (132, 133). Laboratory abnormalities include lymphopenia, neutropenia, anemia, dyslipidemia, and elevated liver enzymes; nonsignificant elevations of creatine phosphokinase and serum creatinine have also been reported (133). However, increased risks of serious infectious episodes, herpes zoster, tuberculosis, opportunistic infections were noted (133). Moreover, the U.S. FDA has added a black box warning on all approved JAK inhibitors indicated for the treatment of arthritis and other inflammatory conditions as well as those approved for AD, namely topical ruxolitinib, oral upadacitinib, and oral abrocitinib (95, 102, 127, 134). The black box warnings included serious infections, mortality, malignancies, major adverse cardiovascular events (MACEs), and thrombosis (95, 102, 127, 134).

Overall, apart from serious infections, other serious adverse events of JAK inhibitors were mostly reported in treating diseases other than AD, such as rheumatic arthritis (95, 102, 127, 134, 135). Among all immune-mediated inflammatory diseases, AD is generally considered as a subgroup with relatively less complicated comorbidity compared to other inflammatory conditions. The underlying inflammation of the disease may contribute to the further adverse events of the treatment (136). For example, regarding the risk of venous thromboembolism, several inflammatory diseases including psoriatic arthritis, rheumatic arthritis, and inflammatory bowel disease, but not AD are proved to be associated with higher risk of venous thromboembolism (137–139). Similarly, among all patients treated with JAK inhibitors, adverse events of venous thromboembolism were mostly reported by patients with rheumatic arthritis other than patients with AD (135). A recent systematic review and meta-analysis also provided the evidence that no increased risk of venous thromboembolism was detected in patients with AD treated with JAK inhibitors (140).

The black box warnings of JAK inhibitors including malignancies and MACEs were based on the ORAL Surveillance study of tofacitinib versus TNF-α inhibitors in rheumatoid arthritis (141). The patients with current or past smokers presented higher risk of malignancies and MACEs. In the population of AD, no significantly increased risk of malignancies or adjudicated MACEs were observed in patients treated with oral abrocitinib, oral upadacitinib, oral baricitinib and topical ruxolitinib in comparison to the control group (87, 106, 111, 115). Nevertheless, sporadic cases non-melanoma skin cancers have been reported in oral upadacitinib, oral baricitinib, oral abrocitinib and topical ruxolitinib (87, 106, 111, 115). Periodic skin examination is suggested for patients with increased risk of skin cancers. Further studies are warranted to evaluate the long-term safety of these JAK inhibitors for AD and their association to serious adverse events listed in black box warning.

Despite the less evidence proving the risk of malignancies and MACEs in patients with AD treated by JAK inhibitors, serious infection due to bacterial, viral, mycobacterial, and opportunistic infections should be always kept in mind in patients with AD treated with JAK inhibitors. Olivera et al. conducted a systematic review and meta-analysis revealed patients with various immune-mediated diseases treated by JAK inhibitors demonstrated higher risk of serious infection, especially herpes zoster (142). The most common serious infection of oral abrocitinib for AD were herpes simplex, herpes zoster, and pneumonia (127). As for oral upadacitinib for AD, the most frequent serious infections were reported as pneumonia and cellulitis (102). Serious lower respiratory tract infections have been reported in clinical studies evaluating the safety of topical ruxolitinib in treating AD (95).

Prior to treatment initiation of systemic JAK inhibitors, evaluations of tuberculosis infection, viral hepatitis, complete blood count, liver function tests, and lipid panel are recommended. Most patients do not require adjustment to therapy based on the laboratory findings (87, 106, 111, 115). Closely monitor patients for the signs of infection, and laboratory abnormalities and interrupt the treatment if necessary (87, 106, 111, 115). Additionally, JAK inhibitors are suggested not to be given in combination with biologic disease-modifying antirheumatic drugs or potent immunosuppressants such as cyclosporine, azathioprine and methotrexate (87, 106, 111, 115). Before the treatment, share decision making is suggested by discussing with the patients about the current evidence of efficacy and safety in order to tailor the optimal treatment strategy (134).

## 4 Conclusions

JAK–STAT signals play a pivotal role in the pathogenesis of AD. In addition to the involvement of various cytokine signaling pathways associated with mainly Th2, as well as Th1, Th17, and Th22 immune responses in AD, JAK–STAT is also engaged in the regulation of the skin barrier and the modulation of peripheral nerves related to the transduction of pruritus of AD. Oral and topically administered novel JAK inhibitors are now being used to treat patients with AD. Notably, topical ruxolitinib, oral upadacitinib, oral baricitinib and oral abrocitinib were approved for the treatment of AD. Considering the balance between efficacy and safety, the choice of the appropriate JAK inhibitors should be made in accordance with the individual patient’s condition. Further studies comparing the efficacy among conventional treatments, biologics, and all small molecules for AD are warranted, and the long-term safety of JAK inhibitors should also be investigated.

## Author contributions

Conceptualization, I-HH, P-CW and C-BC; methodology, I-HH and C-BC; investigation, I-HH, P-CW and C-BC; resources, W-HC and C-BC; writing—original draft preparation, I-HH and C-BC; writing—review and editing, I-HH, P-CW and C-BC; visualization, I-HH; supervision, W-HC and C-BC; project administration, W-HC and C-BC. All authors have read and agreed to the published version of the manuscript. 
